# Shift Work as a Cardiovascular Disease Risk Factor: A Narrative Review

**DOI:** 10.7759/cureus.41186

**Published:** 2023-06-30

**Authors:** Ryan Wong, Alex Crane, Jay Sheth, Harvey N Mayrovitz

**Affiliations:** 1 Medicine, Nova Southeastern University Dr. Kiran C. Patel College of Osteopathic Medicine, Davie, USA; 2 Medicine, University of Pittsburgh School of Medicine, Pittsburgh, USA; 3 Medical Education, Nova Southeastern University Dr. Kiran C. Patel College of Allopathic Medicine, Davie, USA

**Keywords:** shift work, cardiovascular, coronary heart disease (chd), heart, atherosclerosis

## Abstract

Shift work has emerged as a significant health concern in recent years, and research has revealed a link to circadian rhythm dysregulation and atherosclerosis, both of which can increase the risk of cardiovascular disease (CVD). Currently, there is a lack of updated reviews regarding the impact of shiftwork on CVD. Thus, the present narrative review aims to provide a comprehensive summary of the latest research on the relationship between shift work and CVD, identify potential gaps in the current knowledge, and highlight areas for future research. Database searches for peer-reviewed articles published between January 2013 to January 2023 on shift work associated CVD revealed many studies that found shift work is linked with increased prevalence of carotid artery plaque, increased arterial stiffness, and carotid artery intima-media thickness (IMT) all suggestive of a progression of atherosclerosis attributable to shift work. Hypertension, diabetes, and a sedentary lifestyle are known risks for CVD, and the results of the present study suggest that shift work should be added to that list. The elevation of inflammatory markers and DNA damage in shift workers may be linked to their increased progression of atherosclerosis and the positive association of shift work with coronary artery disease. There are minimal studies on mitigating approaches for shift work-related CVD, such as diet modification or exercise, emphasizing the need for further directed research in this area.

## Introduction and background

Shift work involves a work schedule in which employees work during unconventional hours, such as overnight or on weekends, and often involves rotating or alternating shifts. It is a widespread practice in many industries that have been increasingly associated with burnout and recognized as having a potentially harmful impact on cardiovascular health [1-3]. Atherosclerosis, the thickening and hardening of arteries due to plaque development is a major risk factor for the development of coronary heart disease (CHD), a leading cause of death globally killing roughly 375,000 people in 2021 [4]. While lifestyle factors such as physical inactivity, poor diet, and smoking are well-known contributors to atherosclerosis and CHD, the specific relationship between shift work and these conditions is less clear [5-8].

Recently, a growing body of literature has sought to investigate the association between shift work and atherosclerosis and CHD. Studies have suggested that shift work may increase levels of oxidative stress and inflammation, which are thought to contribute to the development of these conditions [9,10]. Additionally, shift work performed by airline pilots and nurses has been linked to disrupted sleep patterns and decreased physical activity, both of which are known risk factors for atherosclerosis and CHD [6,11,12].

The potential mechanisms by which shift work may contribute to atherosclerosis and CHD are varied and involve alterations in cardiovascular risk factors such as elevated blood pressure (BP) and lipid levels, and upregulated pro-inflammatory markers [13-15]. Studies have reported that shift work is associated with increased levels of pro-inflammatory markers, including c-reactive protein (CRP) and interleukin-6 [9,10]. These markers have been linked to increased oxidative stress and disrupted circadian rhythms, both of which are associated with an increased risk of cardiovascular disease (CVD). The impact of shift work on cardiovascular health may also be related to chronobiological mechanisms. Circadian rhythms are biological processes that occur over a 24-hour cycle and play an important role in regulating various physiological processes, such as cardiovascular function [16]. Shift work has been linked to disrupted circadian rhythms, which may lead to DNA damage in cancer progression, and alterations in cardiovascular risk factors associated with a higher risk of developing atherosclerosis and CHD [12,17,18].

However, despite an increasing amount of research examining shift work and atherosclerosis and CHD, a more thorough investigation is needed to fully understand the underlying mechanisms that may potentiate these pathologies. It is also important to note that the impact of shift work on cardiovascular health may be influenced by various factors, such as the type of shift work, the duration of shift work, and the age at which shift work is initiated. Thus, a better understanding of the relationship between shift work and cardiovascular health may help industries implement strategies to mitigate the harmful impacts of shift work and improve the overall health and well-being of their employees. Additionally, the invaluable contributions of stakeholders and policymakers remain crucial in addressing this matter and shaping future initiatives.

## Review

The aim of this narrative review is to provide a comprehensive overview of published literature that discusses the impact of shift work on the cardiovascular system. The goal is to highlight how shift work affects atherosclerosis and CHD risk and discuss the role of diet and exercise as a means to reduce the negative cardiovascular effects associated with shift work.

Search strategy

Three databases were used to identify peer-reviewed articles: PubMed, CINAHL, and Web of Science. One or more of the following phrases was mandatory in the title or abstract: “night shift”, “nightshift”, “rotating shift”, “shift work”, and “shiftwork”. Additionally, one or more of the following words was mandatory in the title or abstract: “coronar*”, “atherosc*”. Studies published between January 2013 and January 2023 were included in the present review. A total of 182 articles were identified that met the inclusion criteria. These articles were then filtered by the following exclusion criteria: review articles, poster abstracts, animal studies, non-English articles, articles with only abstracts available, commentaries and editorials, and articles with non-atherosclerosis and non-coronary artery disease focus (ie. impact of shift work on clinical decision making in coronary care, spectral analysis of atherosclerotic plaques, the economics of coronary care, etc.). After filtering, 35 articles remained, which were evaluated and presented in this review.

The impact of shift work on atherosclerosis

Shift work has been reported to be linked to the dysregulation of circadian rhythms characterized by insomnia and/or amplified sleepiness, particularly in nurses and airline pilots [19,20]. A study found that shift workers with insomnia had a 5.8% greater carotid plaque prevalence compared to non-insomnia shift workers [21]. These findings were based on carotid artery ultrasound determinations. A study of 110 male chemical plant workers found that shift work employment was linked to a 2.89-fold increase in coronary plaque development [22]. These findings were based on measures of coronary artery calcium score via coronary artery computed tomography (CT) angiography in shift workers vs. day workers. Another report assessed subclinical atherosclerosis in the carotid or femoral arteries defined as the presence of a protruding focal plaque that measured at least 0.5 mm or > 50% thicker than the surrounding intima-media thickness (IMT) [23]. In this study, a total of 2459 Spanish men, aged 39-59 years, were evaluated. Workers performing morning/evening/night rotation shifts had an increased odds ratio (OR) of atherosclerosis (OR: 1.46; 95% confidence interval (CI): 1.02, 20.9) (p < 0.05) vs. groups working solely nights, rotating morning/evening, or central shifts. Additionally, a carotid ultrasound examination of 3582 Chinese steelworkers (mean age = 46 years) found that working night shifts for greater than 20 years revealed significant elevation of carotid plaque presence compared to those who worked less than 20 years [24]. Age, a potential compounding factor was considered a covariate along with others such as hypertension, diabetes, and body mass index (BMI).

Other clinical measures have been used to assess the risk of atherosclerosis in shift work. One study looked at the atherogenic index, a measure of triglycerides and high-density lipoprotein cholesterol ratio, in 140 healthy Jordanian hospital day and night shift employees (males and females, ages 20-59) with no previous CVD at baseline. The atherogenic index was found to be higher in night shift workers compared to daytime workers (p = 0.024) [25]. Another examined advanced atherosclerosis in 10,475 participants aged 35-64 from the Gutenberg Health Study, a population-based cohort study in Germany [1]. Subclinical atherosclerosis was measured by two parameters. One was arterial stiffness determined by pulse wave analysis. The other was the reactive hyperemia index (RHI), a measure of vascular function, determined by volume plethysmography. Their results showed that a greater number of cumulative night shift exposures were associated with elevated arterial stiffness. Night shift work, as opposed to non-night shift work was correlated with a significant decrease in RHI, indicating an increased risk of atherosclerosis. A cross-sectional study in Japan investigated the accumulation of visceral fat area (VFA) measured by CT and a cardio-ankle vascular index (CAVI) as a measure of atherosclerosis in 10,073 middle-aged male shift workers (46.6 ± 8.1-year-old) [26]. CAVI is measured by cardio-ankle pulse wave velocity; an increased pulse wave velocity indicates stiffer arteries. They found significantly less VFA (p < 0.0001) and a smaller CAVI value (p < 0.01) in 3838 shift workers compared to 6235 fixed daytime workers. These findings would seem to indicate that the shift workers were in better physical shape with less stiff arteries than the non-shift workers. However, it is important to note that 91.2% of shift workers in this study performed physical work as opposed to office work. 68.4% of fixed daytime workers in this study performed office work as opposed to physical work. Thus, a major limitation of this study was failing to control for the type of work performed in determining a direct correlation between shift work, VFA, and CAVI.

Several studies have examined carotid artery IMT as a measure of atherosclerosis progression. A study that involved 94 industrial plant workers found a positive association between carotid artery IMT and years of shift work (p = 0.009) after adjusting for age, sex, and smoking (pack years) as possible confounders [27]. A recent study from Japan also indicated greater carotid artery IMT in shift workers (p < 0.01) [26]. A study of data from the Brazilian Longitudinal Study of Adult Health found that among 886 men, a one standard deviation increase in years of night shift work was associated with higher carotid IMT [28]. In contrast, this study found no association between years of night shift work and carotid IMT in 892 women. This finding is consistent with that of a cross sectional survey that found a lack of significant association between night shift work and carotid IMT after adjusting for confounding factors such as sleep duration, insomnia, BMI, hypertension, and diabetes [29]. Another study of male South African truck drivers, aged ≥ 18 years (n = 607) working day shift only or night and day shift, found that atherosclerotic CVD risk score and carotid IMT were not associated with night and day shift work [5]. These findings reveal divergent results regarding carotid IMT and shift work, suggesting that the variability could potentially stem from the inherent proficiency and skill set of the operator responsible for obtaining the measurements.

The impact of shift work on coronary heart disease

Atherosclerosis is one of the major causes of CHD. Plaque deposition results in stenosis or occlusion of the coronary arteries which can lead to various forms of CHD, including angina, myocardial infarction (MI), and arrhythmias. In 2020, CHD was the most common type of heart disease [30].

Several studies have investigated the association between shift work and CHD. One 24-year prospective cohort study found that women (n = 73,623) who worked ≥ 10 years of shift work had a significantly elevated CHD risk [95% confidence interval (CI): 1.04-1.24, p = 0.004)] compared to those who never worked night shifts [31]. A more recent prospective study that surveyed retired workers (n = 21,802) after a five-year follow-up also reported similar findings [32]. In an analysis of hazard ratios (HR) as a measure of CHD risk of retired persons, workers with > 20.00 years of past shift work had higher HR compared to those that worked < 5 years of past shift work (HR: 1.28, 95% CI: 1.08 - 1.51 vs. HR: 1.05, 95% CI: 0.94 - 1.16). Furthermore, a study from the UK Biobank reported that working greater than 10 years of night shift (n = 6640) was strongly associated with a greater risk of CHD (HR: 1.37, 95% CI: 1.20-1.58) [33]. Individuals working three to eight nights per month of night shift (n = 7550) also had increased CHD risk (HR: 1.35, 95% CI: 1.18-1.55). Notably, when age was considered a covariate, atrial fibrillation risk remained elevated in those who worked three to eight nights per month (HR: 1.22, 95% CI: 1.02-1.45).

A major contributor to CHD is coronary artery disease (CAD) defined as pathological plaque accumulation in coronary arteries that can be either nonobstructive or obstructive and potentiate into CHD [34]. While often used interchangeably, CHD typically refers to the clinical manifestation of a patients’ CAD. A study from Israel utilized CT angiography to evaluate CAD risk in 94 shift workers and 255 non-shift workers [35]. The extent of CAD tended to be higher in shift workers but was not statistically significant (p = 0.06). However, in that study, the presence of ­­­coronary artery stenosis > 50% was more prevalent in the shift workers (p = 0.006). Another study predicted CAD risk in Iranian nurses working rotating shifts by utilizing the Framingham risk score (FRS), a measure of estimating the 10-year risk of manifesting clinical CVD [15]. They found that the FRS of these shift workers was higher compared to non-shift workers. FRS data also revealed an increased prevalence of CAD risk in shift work (p = 0.04). Moreover, total cholesterol and BP were also significantly higher in shift workers (p ≤ 0.001). A study me­­­­­­ntioned previously on Jordanian hospital workers also found that there was an increase in FRS, reported by the authors as p = 0.000 [25].

Work stress as a factor in CHD progression

Work stress and burnout as consequences of shift work have been reported to be important contributors toward adverse cardiovascular health [3]. A study of all New Zealand residents aged 20-64 years assessed the risk of ischemic heart disease associated with long working hours and night shift work [36]. This study was done by the analysis of a New Zealand longitudinal meta-dataset. It was reported that night shift work was associated with increased ischemic heart disease risk (HR: 1.10; 95% CI: 1.05 to 1.14 and HR: 1.25; 95% CI: 1.17 to 1.34) in both males and females respectively. Interestingly, an 8-year longitudinal study of female nurses found that those who engaged in shift work were more likely to have daytime sleepiness almost every day. However, daytime sleepiness was not the sole contributor to CHD and CVD [6]. Variables such as sleep characteristics and cardiometabolic risk were critical in promoting the incidence of CVD, stroke, and CHD. Thus, this study indicates that shift work may synergistically work with covariates in CHD progression. 

Inflammatory markers in shift work associated atherosclerosis and CHD progression

There is evidence that shift work is associated with an increased risk of atherosclerosis and its complications including CHD. Since both pathologies have inflammatory components, it is of interest to examine the potential impact of shift work on inflammatory markers. Several biomarkers have been proposed to explain these pathological outcomes. CRP and homocysteine are known to be risk factors associated with CVD and metabolic syndrome [37,38]. A Taiwanese study measured the sensitivity of CRP and homocysteine in the blood plasma of 825 long-haul bus drivers working in shifts and reported that drivers ≤ 35 years old had increased CRP and homocysteine levels as compared to those who were older than 35 [10]. These inflammatory markers were attributed to increased job strain within this cohort which demonstrates the importance of decreased rest and increased physical inactivity as stressors on shift work-driven inflammation. A more recent experimental study looked at 60 resident physicians aged 25-35 working 24-hour shifts [39]. CRP was measured via phlebotomy and found to be significantly greater in individuals after a 24-hour shift compared to those after an eight-hour working shift (1.57 mg/L vs. 0.79 mg/L) (p = 0.024). These findings highlight occupational stress as a proinflammatory mediator. Finally, a comparative study that looked at rotating night shift workers (n = 88) versus day workers (n = 64) found that CRP was not only increased in the shift workers (p = 0.025), but interleukin-1β (IL-1β) was also higher in rotating night shift workers (p = 0.043) [9]. In this study, anthropometric parameters such as carotid IMT, glucose, and lipids were also found to be greater in rotating-night shift workers, suggesting subclinical atherosclerotic process association with shift work. Additionally, glucose and lipid levels may provoke metabolic syndrome which incites adverse cardiovascular events [40].

Further exploration into the impact of shift work encompassing not only inflammation but also oxidative stress and DNA damage, presents a promising avenue for targeted research. Studies have shown that impaired DNA repair mechanisms are implicated in the pathogenesis of chronic diseases like cancer and diabetes [18]. Additionally, DNA damage plays a significant role in vascular aging, atherosclerosis, and calcification [41]. DNA damage has been shown to induce apoptosis and vascular cell senescence. In conjunction with current studies on DNA damage provoked metabolic alterations secondary to shift work, there is important room for directed research looking at DNA damage and oxidative stress in shift work-related cardiovascular health.

Sleep and hypertension in shift work-related cardiovascular health

A multinational double-blind, placebo-controlled trial examined obstructive sleep apnea, shift work, and major coronary events as defined by CHD death, MI, or urgent revascularization in participants with ≤ 30 days of acute coronary syndrome (n = 13,026) [42]. In that study, it was found that less than six hours of sleep was associated with a 29% higher risk of major coronary events compared to those with longer sleep (p < 0.001). In addition, persons who worked overnight shifts for ≥ 3-night shifts per week for ≥ 1 year, had 15 % higher risk of major coronary events (p = 0.01) after a 2-year follow-up. This study concluded that overnight shift work may be considered an independent risk factor for adverse cardiovascular outcomes.

A significant risk factor for CVD is arterial hypertension [43]. A cohort study of the UK Biobank database examined whether shift workers with hypertension were at risk for progression to cardiometabolic multimorbidity (CMM), defined as the presence of hypertension and one of the following: diabetes, CHD, or stroke [44]. The authors found “usually, or always” working night shifts was associated with a 16% higher risk of CMM compared to working day shifts (HR: 1.16, p = 0.025). Further, working more than 10 night shifts per month was associated with a 19% greater risk of CMM (HR: 1.19, p = 0 .005). Both risk models adjusted for possible covariates such as age, sex, race, BMI, and social history. Thus, in persons with hypertension, night-shift work may be considered a risk factor for CMM. A separate study looked at the BP during sleep of 30 female medical workers after performing a day shift or a night shift. BP was measured each hour during sleep and they found that the standard deviation of diastolic BP was greater in post-night shift workers compared to that of post-day shift workers (p < 0.01) [14]. The increase in diastolic BP variability could be a risk factor for CVD in young people since it was previously reported that nighttime BP variability is associated with increased cardiovascular mortality (p = 0.002) [45].

Lifestyle alterations in reducing adverse cardiovascular outcomes in shift workers

Diet and exercise are life-style choices that can help mitigate CVD risk [8]. A pilot randomized controlled study of Australian night shift workers (n = 19) examined the effects of an acute meal challenge on metabolic markers of CVD risk after completion of a control period or intervention period, during which they fasted nightly for five hours [46]. After the fasting period, participants had significantly lower body weights (mean difference -0.9; 95% CI: -1.3 to -0.4; p < 0.001), without any differences in energy expenditure. There were no differences in peak post-prandial glucose or triglyceride levels. The difference in body weight is thought to be a result of the differences in the thermic effect of food between daytime and nighttime eating [47].

There are several studies that have examined physical inactivity and its impact on shift work-related cardiovascular health. One study looked at the influence of shift work on BMI, waist-hip ratio, smoking, sedentary lifestyle, elevated BP, hyperlipidemia, and type II diabetes among first-time MI cases of men and women (aged 45-70; n = 4648) [13]. The synergy index (SI) (SI = 1 = no interaction; SI > 1 = positive interaction; SI < 1 = negative interaction) was used to compare shift work and CVD risk factors in men and women. Physical inactivity and shift work promoted MI for male shift workers (SI = 2.05, 95% CI). For female shift workers, a high waist-hip ratio (SI = 4.0, 95% CI) and hyperlipidemia (SI = 5.69, 95% CI) interacted with the risk of MI. Moreover, time spent in a sedentary posture was correlated with greater CHD risk. Furthermore, a cross-sectional study (n = 111) examining the association between time spent in a sedentary posture of shift workers and CHD risk found that the latter was associated with greater triglycerides, lower high-density lipoproteins, and larger waist circumference (p = 0.002, p = 0.001, p = 0.005, respectively) [48]. Sedentary time was also associated with a higher 10-year prospective cardiovascular risk as calculated using the Prospective Cardiovascular Münster (PROCAM) risk score (p = 0.047). PROCAM is a 10-year CHD risk calculator for men aged 35-65 years and women aged 45-65 years [49].

It is well-established that exercise is a lifestyle modification that improves cardiovascular health [50-53]. A study of Norwegian factory shift workers (n = 65) found that a regimen of 17 minutes of high-intensity exercise, three times a week for eight weeks significantly improved aortic systolic and diastolic BP as well as glycated hemoglobin (HbA1c) [54]. Baseline and post-intervention measurements were acquired for 19 participants in the intervention group who attended >10 training sessions. Both aortic systolic and diastolic BP significantly decreased from baseline in the intervention group (p < 0.05). HbA1c was also significantly lower (p < 0.01). A separate randomized controlled study of night shift workers (n = 30) also found that intermittent exercise reduced biomarkers of atherosclerosis [55]. Intermittent exercise was defined as 30 minutes of brisk walking to maintain a heart rate between 60% and 79% of maximal heart rate, three times weekly. After 10 weeks, participants (n = 15) randomized to the exercise group had greater decreases in serum concentration of Cathepsins S and L, soluble E-selectin, and soluble vascular cell adhesion molecule-1 when compared to the responses of the control group (p < 0.01). There were no significant differences found in BP measurements. Thus, for shift workers with variable schedules, short periods of intermittent exercise may reduce CHD risk.

## Conclusions

An analysis of the evaluated literature provides compelling evidence that supports the association between shift work and an increased risk of atherosclerosis and CHD. Several studies examined atherosclerotic progression by employing carotid IMT as a parameter, indicating its reliability for assessment. However, it is worth mentioning that within the reviewed studies, three found no association between shift work and carotid IMT, which may be attributable to the omission of confounding factors in their analyses. The risk of atherosclerosis and CHD appears to escalate with the duration of shift work exposure indicating a progressive onset. It would be valuable to conduct additional research focused on a younger age cohort to explore the long-term cardiovascular outcomes associated with shift work. Moreover, as remote working gains prevalence, it becomes crucial to examine how irregular sleep-wake cycles and disrupted sleep patterns, in the context of modified remote work arrangements, influence cardiovascular health. Additionally, the reviewed research indicates that shift work is linked to elevated levels of inflammatory markers such as CRP, homocysteine, and IL-1β. Lifestyle modification such as nightly fasting for five hours does not significantly reduce metabolic markers of CVD. Thus, further research is needed to elucidate the role of irregular eating patterns on CVD progression of shift workers. Two reviewed studies found that physical activity in shift workers was beneficial for their cardiovascular health. Incorporating high-intensity training and occasional exercise may serve as significant lifestyle modifications to improve the cardiovascular well-being of individuals engaged in shift work. Emphasizing the need for interventions and policies aimed at reducing risks among shift workers is necessary, as it can contribute to improving occupational settings and cardiovascular health.

Taken together, this review highlights the current research regarding shift work as a potential work hazard by impacting cardiovascular health and indicates that significant opportunities exist for future research in this field. The information provided herein may help understanding of links between shift work and cardiovascular health and assist in developing interventions to improve occupational conditions.
